# Idiopathic Left Ventricular Pseudoaneurysm: A Case Report Highlighting Its Rare Occurrence and Management

**DOI:** 10.7759/cureus.78615

**Published:** 2025-02-06

**Authors:** Shunya Ono, Motoharu Shimozawa, Retsu Tateishi, Kosaku Nishigawa, Takeyuki Kanemura

**Affiliations:** 1 Cardiovascular Surgery, IMS Katsushika Heart Center, Tokyo, JPN

**Keywords:** diagnostic images, idiopathic left ventricular pseudoaneurysm, late gadolinium enhancement, pericardial patch repair, surgical adhesives

## Abstract

Left ventricular (LV) pseudoaneurysms are rare and life-threatening conditions caused by myocardial rupture contained by external tissues. Idiopathic cases, with no identifiable cause, are exceptionally rare. We report the case of a 45-year-old asymptomatic male with no significant medical or family history, in whom an incidental LV pseudoaneurysm was discovered during a routine health check. Imaging revealed a 30×29×21 mm pseudoaneurysm with thin walls and active blood flow, posing a high risk of rupture despite the absence of symptoms. Preventive surgical repair was performed using a bovine pericardial patch reinforced with sutures, Teflon felt strips, and surgical glue. Postoperative recovery was uneventful, and follow-up imaging confirmed successful repair with no recurrence. This case highlights the rarity of idiopathic LV pseudoaneurysms and the importance of early diagnosis and intervention to prevent fatal outcomes.

## Introduction

A left ventricular (LV) pseudoaneurysm is a rare but dangerous condition, defined as a contained myocardial rupture that lacks a complete ventricular wall and relies on external tissues for structural support [1]. Unlike true aneurysms, pseudoaneurysms are structurally weaker and carry a higher risk of rupture [2,3]. They are most often associated with myocardial infarction as well as cardiac surgery, trauma, or infections [1]. Idiopathic cases, where no clear cause can be identified, are exceedingly rare [4]. Diagnosis typically involves imaging techniques, such as echocardiography, computed tomography (CT), or magnetic resonance imaging (MRI), to identify the characteristic thin walls and narrow neck of the pseudoaneurysm [1].

This report highlights the case of a 45-year-old man with no symptoms or identifiable risk factors, in whom an idiopathic pseudoaneurysm was discovered and successfully treated with patch repair, resulting in an excellent outcome.

## Case presentation

The patient was a 45-year-old male with a medical history significant only for hyperlipidemia, for which he was taking statin therapy. He had no known comorbidities, no family history of cardiovascular disease, or sudden cardiac death. The patient reported no symptoms, had a New York Heart Association (NYHA) functional classification of I, and consumed alcohol only occasionally. During a routine health check, he was found to have mildly elevated liver enzymes and underwent a CT scan of the abdomen, which incidentally revealed a pseudoaneurysm in the LV wall. He was subsequently referred to our hospital for further evaluation and management.

**Figure 1:**
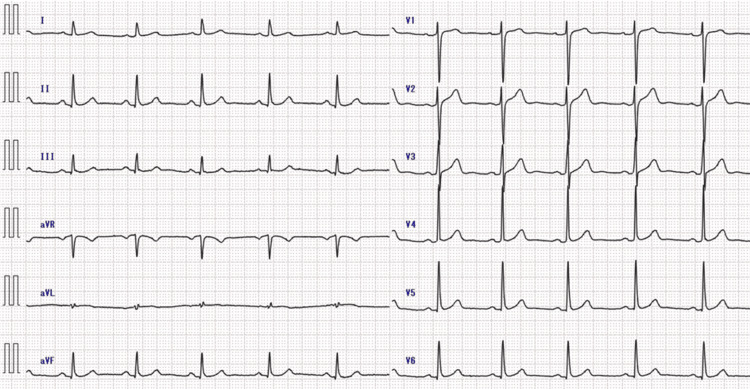
Preoperative electrocardiogram (ECG) No ischemic changes, including ST segment abnormalities, were observed.

At our hospital, detailed imaging tests were performed. Electrocardiogram (ECG) showed normal sinus rhythm with no ischemic changes such as ST-segment abnormalities (Figure 1).

**Figure 2:**
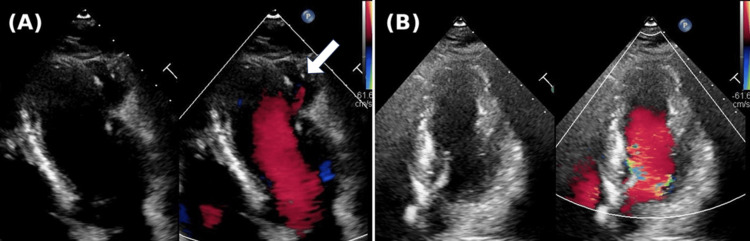
Echocardiographic images showing the left ventricle in diastole (A) Preoperative echocardiography. Blood flow entering the pseudoaneurysm is observed (white arrow). (B) Postoperative echocardiography. The pseudoaneurysm is completely sealed, and no leakage is detected.

**Figure 3:**
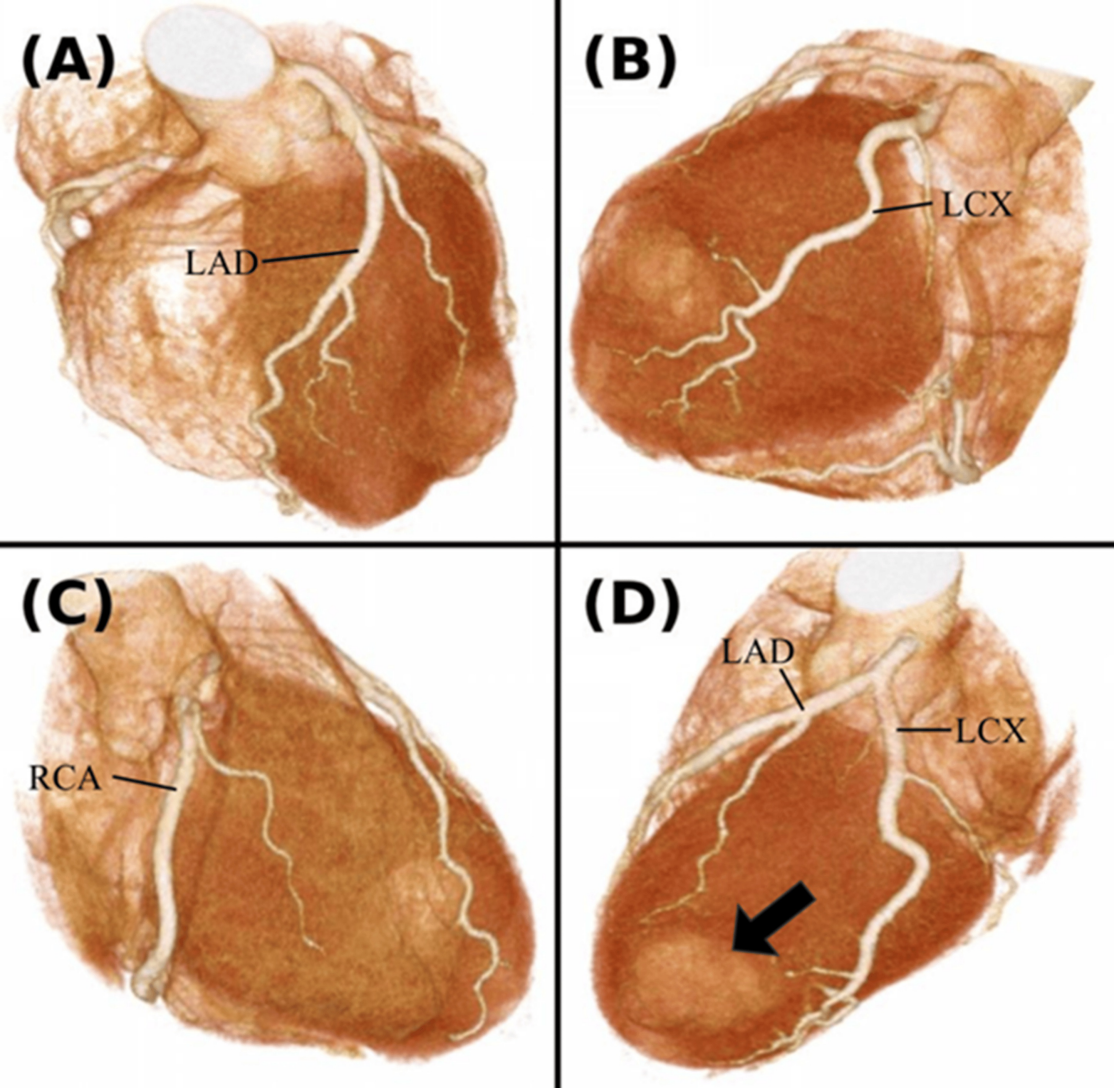
Preoperative contrast-enhanced cardiac computed tomography (CT) with 3D reconstruction of the coronary arteries (A) Left anterior descending artery (LAD). No stenosis observed. (B) Left circumflex artery (LCX). No stenosis observed. (C) Right coronary artery (RCA). No stenosis observed. (D) A pseudoaneurysm is located between the LAD and LCX (black arrow).

**Figure 4:**
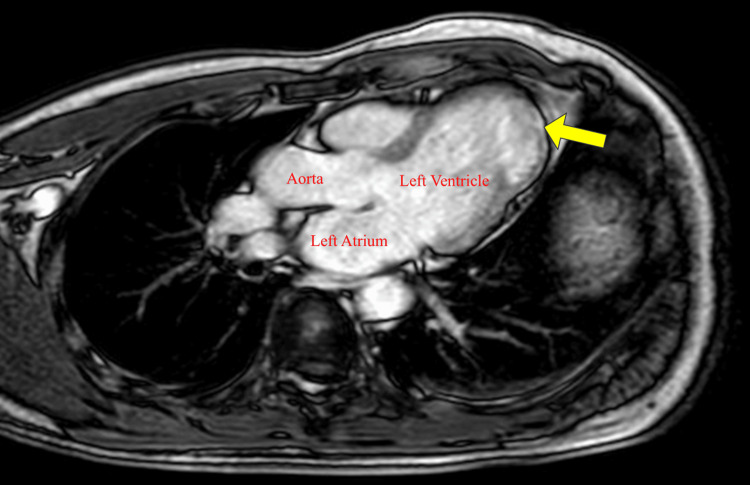
Magnetic resonance imaging (MRI) with late gadolinium enhancement (LGE) Contrast enhancement is observed in the sac of the pseudoaneurysm (yellow arrow).

Echocardiography revealed a pseudoaneurysm located near the LV lateral wall, characterized by a thin wall and blood flow within the sac, without evidence of thrombus. The ejection fraction (EF) was 71%, with akinesis observed only at the site of the pseudoaneurysm while no asynergy was noted in other regions (Figure 2A). Additionally, no significant valvular disease was detected. Contrast-enhanced cardiac CT confirmed the location of the 30×29×21 mm pseudoaneurysm between the left anterior descending (LAD) and left circumflex (LCX) arteries, but no significant coronary artery stenosis was seen (Figure 3). Magnetic resonance imaging (MRI) with late gadolinium enhancement (LGE) revealed contrast uptake in the sac of the pseudoaneurysm (Figure 4). Gallium scintigraphy was negative for signs of sarcoidosis or other inflammatory conditions. Despite the patient being asymptomatic, the pseudoaneurysm was considered at high risk of rupture due to its size, thin walls, and active blood flow. We decided to proceed with preventive surgery to avoid rupture.

**Figure 5:**
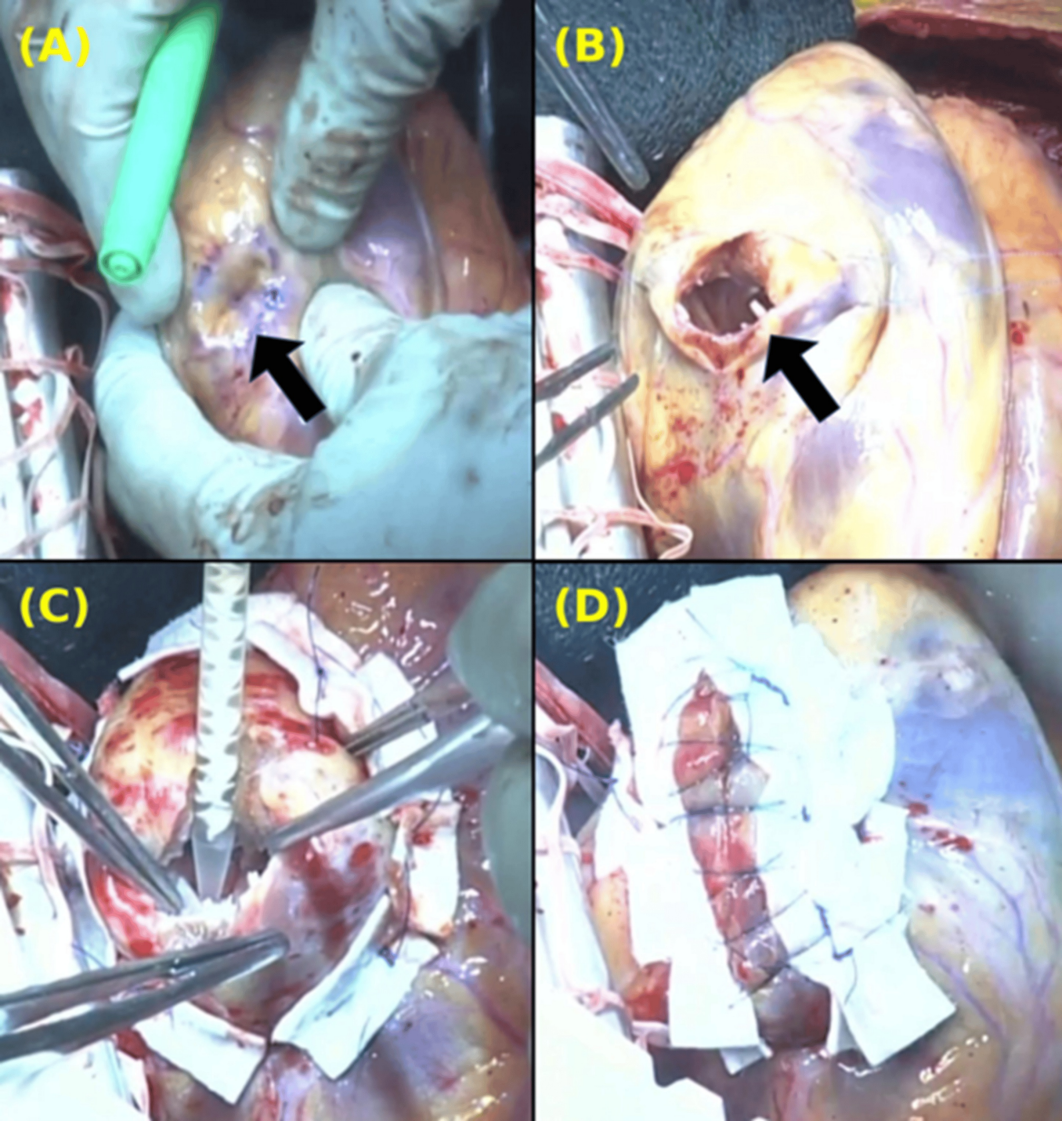
Intraoperative findings The images are oriented with the patient’s head at the bottom and the foot at the top. The right side corresponds to the surgeon’s side, and the left side corresponds to the patient’s left. (A) A pseudoaneurysm with a thinned wall located between the LAD and LCX (black arrow). (B) The pseudoaneurysm was opened and trimmed (black arrow). (C) The defect was closed with a bovine pericardium, and BioGlue was applied to prevent leakage. (D) The closure was reinforced with two Teflon felt strips, using both mattress and continuous sutures. LAD: left anterior descending; LCX: left circumflex

The surgery was performed under general anesthesia. Through a median sternotomy, cardiopulmonary bypass (CPB) was established with aortic and right atrial venous cannulation. An aortic clamp was applied, and cardiac arrest was achieved using antegrade myocardial protection. The heart was mobilized and exteriorized to allow exposure of the pseudoaneurysm. The pseudoaneurysm was located on the apical side of the lateral wall, without any external protrusion, but thinning of the wall was observed (Figures 5A, 5B). A patch made of bovine pericardium was trimmed into a 4 cm diameter regular octagonal shape and secured to the LV wall using 3-0 polypropylene sutures. Eight mattress sutures were placed through the patch and passed from the LV intracavitary side to the external surface of the LV, where they were tied. BioGlue (CryoLife, Inc., Kennesaw, GA, USA), a surgical adhesive composed of bovine serum albumin and glutaraldehyde, was applied between the patch and the ventricular tissue to prevent leakage (Figures 5C, 56). The incision in the left ventricular wall was first reinforced by sandwiching it between two 15-mm-wide Teflon felt strips. It was then closed using 3-0 polypropylene mattress sutures, followed by a two-layer continuous suture closure. Finally, BioGlue was applied for additional reinforcement to enhance sealing and prevent leakage. After weaning off the cardiopulmonary bypass, no bleeding from the suture line was observed. Postoperative transesophageal echocardiography confirmed the absence of leaks or complications, and no new asynergy was observed except for akinesis at the closure site.

**Figure 6:**
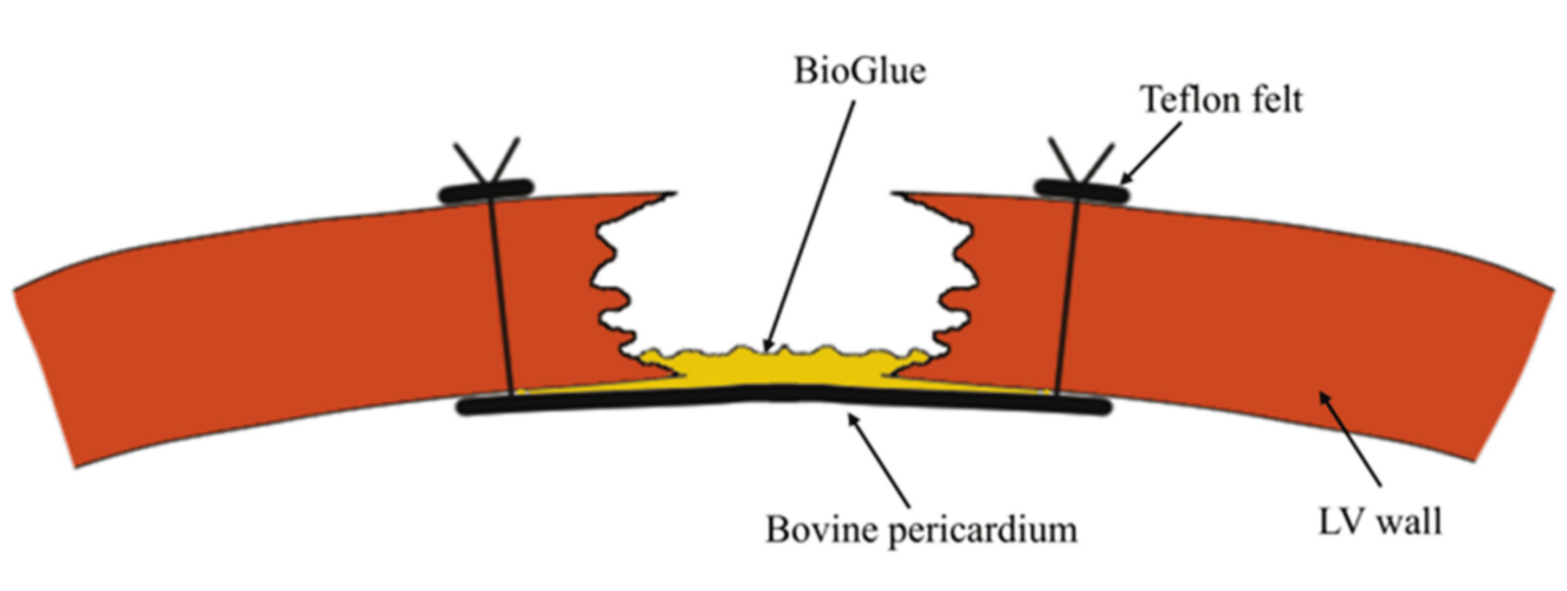
Schematic diagram of patch closure A 3-0 polypropylene suture was passed through the bovine pericardium and secured from the left ventricular cavity to the free wall. BioGlue was applied between the patch and the left ventricle to prevent leakage. Image Credits: Shunya Ono

**Figure 7:**
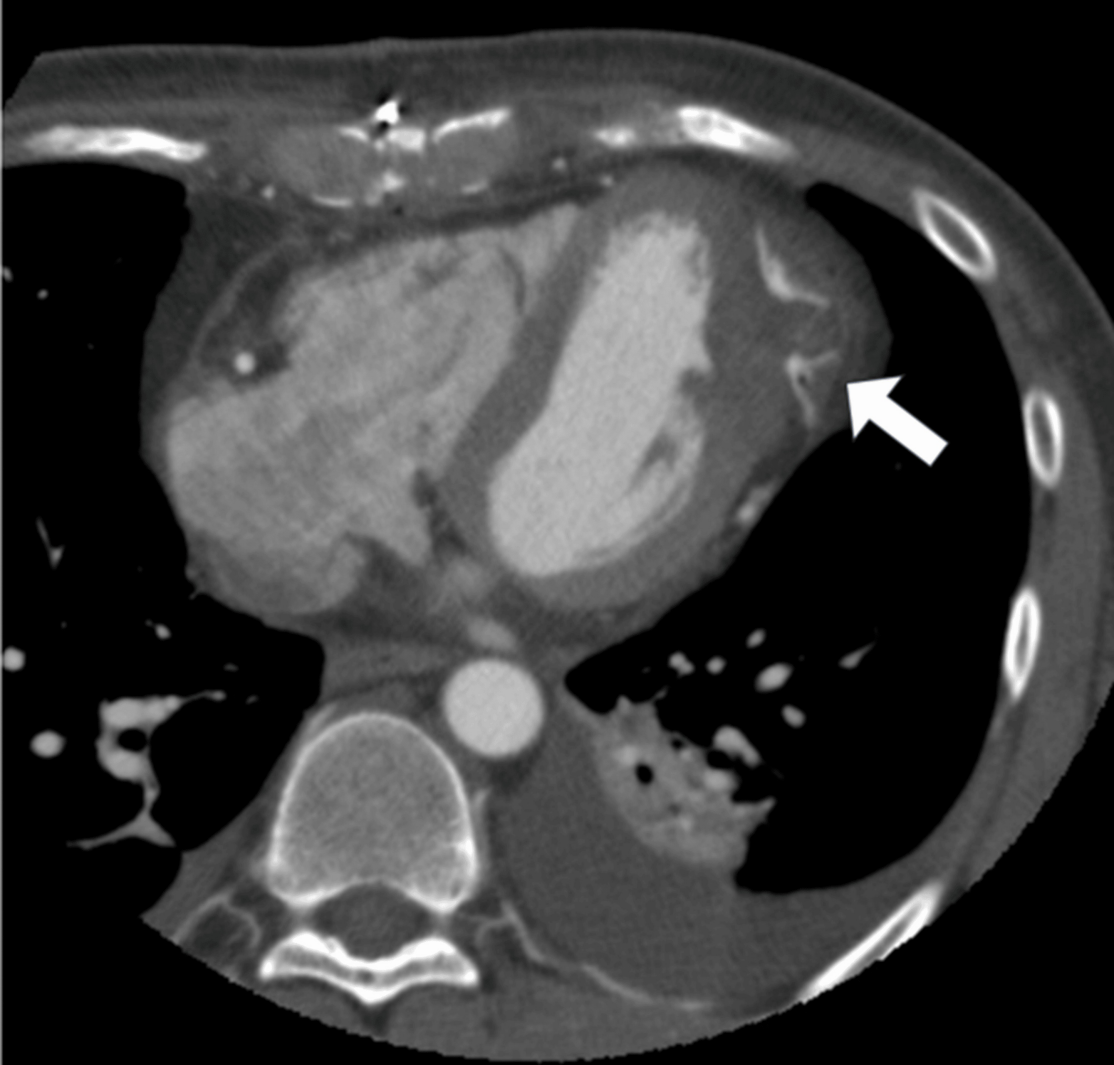
Postoperative contrast-enhanced CT in the axial plane The pseudoaneurysm is completely sealed with no leakage (white arrow). The left ventricular geometry is preserved.

The patient remained hemodynamically stable and was transferred to the intensive care unit, where extubation was successfully performed on the day of surgery without issues. On postoperative day (POD) 5, echocardiography showed preserved EF with no new asynergy, consistent with preoperative findings (Figure 2B). Contrast-enhanced CT confirmed the integrity of the patch closure, with no evidence of leakage (Figure 7). The patient maintained New York Heart Association (NYHA) class I status and was discharged on POD 7 in excellent condition. After discharge, the patient quickly returned to normal daily activities without experiencing any complications, symptoms, or limitations in physical function. No adverse events were reported during the follow-up period.

## Discussion

LV pseudoaneurysms are rare and arise from myocardial rupture contained by the pericardium or scar tissue rather than normal ventricular wall structures. The primary causes include myocardial infarction, cardiac surgery, trauma, infections, and rarely, inflammatory diseases such as sarcoidosis [1]. Unlike true aneurysms, which develop due to the gradual weakening and dilation of all three layers of the ventricular wall, pseudoaneurysms lack a complete wall structure and rely on external tissue for containment, making them more prone to rupture [2,3]. Idiopathic LV pseudoaneurysms, as observed in this case, are exceedingly rare, with very few cases reported in the literature [4].

Diagnosis requires distinguishing pseudoaneurysms from true aneurysms based on imaging features. Echocardiography is often the first-line modality due to its accessibility and ability to detect characteristic features such as thin walls, a narrow neck, and blood flow within the pseudoaneurysm [1]. Computed tomography (CT) provides high-resolution imaging, allowing for precise localization, measurement, and identification of associated coronary artery disease [5]. Magnetic resonance imaging (MRI) offers superior tissue characterization, and late gadolinium enhancement (LGE) plays a key role in differentiating pseudoaneurysms from true aneurysms. In pseudoaneurysms, LGE highlights the fibrotic boundary and thin wall of the lesion, aiding in diagnosis and surgical planning. In this case, LGE confirmed the presence of a pseudoaneurysm by showing contrast uptake in the sac, a feature consistent with previous reports. The ability of MRI to precisely delineate tissue structure makes it indispensable, particularly when other modalities provide inconclusive results [6]. Gallium scintigraphy can further exclude inflammatory conditions, such as sarcoidosis, in atypical presentations [7].

Indications for surgery include the presence of symptoms, a large pseudoaneurysm (≥3 cm in diameter), or imaging features suggestive of imminent rupture, such as thin walls or active blood flow. While some small and asymptomatic pseudoaneurysms may be managed conservatively, surgical intervention is generally recommended due to the high risk of rupture, which can lead to catastrophic outcomes [8,9]. The decision to operate must also consider patient-specific factors, including comorbidities and surgical risk. In asymptomatic cases with high-risk features, such as wall thinning and a size exceeding 3 cm, as seen in this patient, elective surgery is justified to prevent rupture. Early surgical intervention has been associated with favorable outcomes in terms of long-term survival and freedom from recurrence [10].

The surgical management of LV pseudoaneurysms primarily involves patch repair to restore structural integrity while minimizing tension on the fragile ventricular wall. Direct closure, although conceptually simpler, is typically avoided due to the excessive tension it exerts on the weakened ventricular tissue, which increases the risk of suture dehiscence, recurrent pseudoaneurysm formation, or even rupture [11]. Several materials are available for patch repair, including bovine pericardium, synthetic materials (e.g., Dacron or polytetrafluoroethylene (PTFE)), and autologous pericardium. Bovine pericardium is widely preferred due to its favorable handling properties, excellent biocompatibility, and durability [10]. Synthetic patches, while offering durability, carry a higher risk of infection and may lack the flexibility needed to adapt to the myocardial contour [12]. Autologous pericardium, while providing superior biocompatibility, may be limited by its availability or the requirement for additional dissection. The choice of patch material should be determined based on the pseudoaneurysm’s size and location, as well as patient-specific factors, including the presence of infection or a history of previous surgeries. A closure technique utilizing two strips of Teflon felt to sandwich the repair site, combined with mattress and continuous sutures, has been reported as a potential method to reduce bleeding and recurrence [11]. In this case, BioGlue was applied to provide additional reinforcement for enhanced stability. Precise surgical planning and meticulous execution remain pivotal in ensuring favorable outcomes, particularly in anatomically complex or challenging cases.

## Conclusions

This case demonstrates the successful diagnosis and management of an idiopathic LV pseudoaneurysm, a rare and potentially dangerous condition. Despite the patient being asymptomatic, advanced imaging modalities, including echocardiography, CT, and MRI with LGE, were crucial in accurately identifying the pseudoaneurysm and assessing its risk of rupture. Preventive surgical repair using a bovine pericardial patch effectively restored structural integrity and avoided potential complications. This report contributes to the limited literature on idiopathic LV pseudoaneurysms and highlights the importance of careful evaluation and proactive management in ensuring positive outcomes.
